# Supplementary figures and images for: Wildlife overpass structure size, distribution, effectiveness, and adherence to expert design recommendations
Source: PeerJ. 2022 Dec 12;10:e14371. doi: 10.7717/peerj.14371 (PMC9753749; doi:10.7717/peerj.14371)

## Overpass dimensions

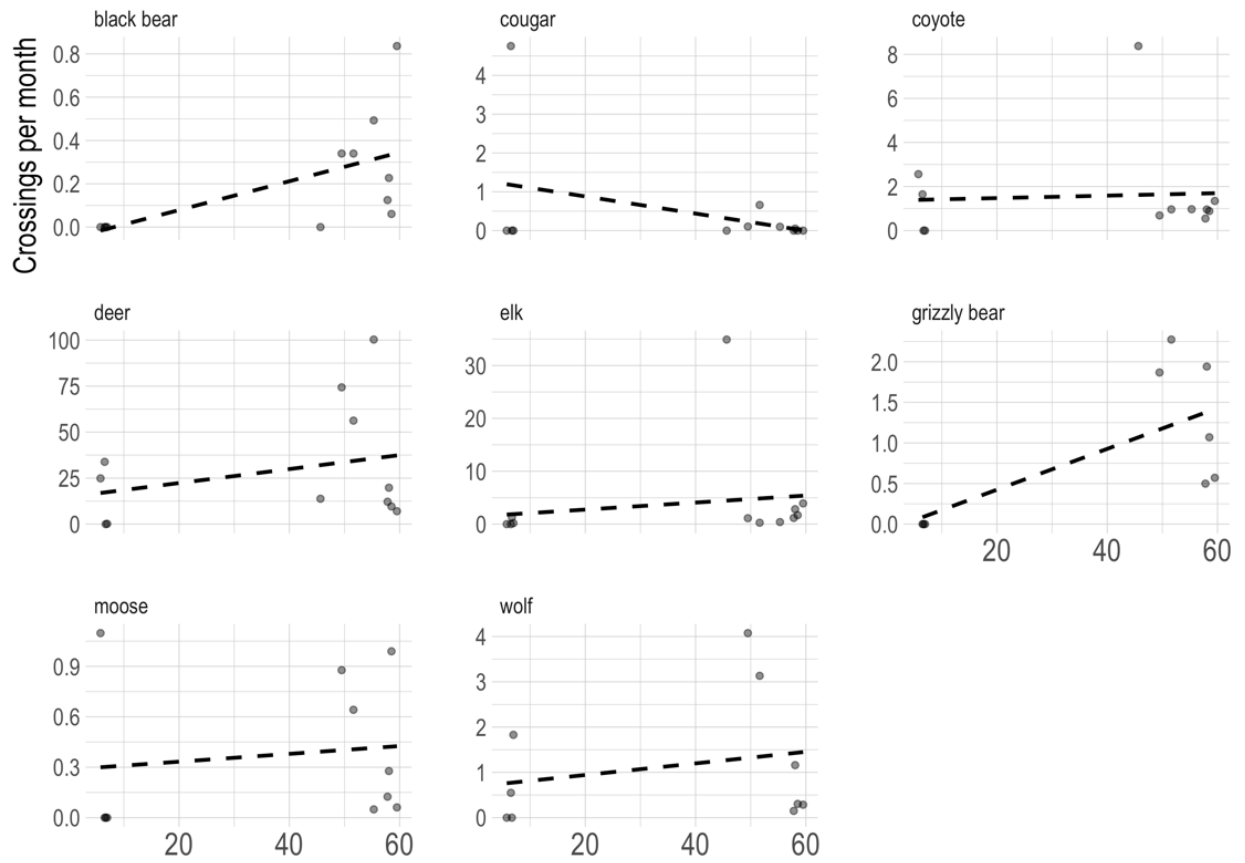

Supplement: Supplemental Information 1 — Dots represent individual overpass structures. Data compiled from transportation agencies and government reports. Species included in analysis: (black bears (Ursus americanus), grizzly bears (Ursus arctos),wolves (Canis lupus), coyote (Canis latrans), cougars (Puma concolor), deer (Odocoileus sp.), elk (Cervus elaphus) moose (Alces alces) crossing rates and the width of 12 overpasses located in western North America. See Supplemental Information Table S7 for details. [file peerj-10-14371-s001.pdf]

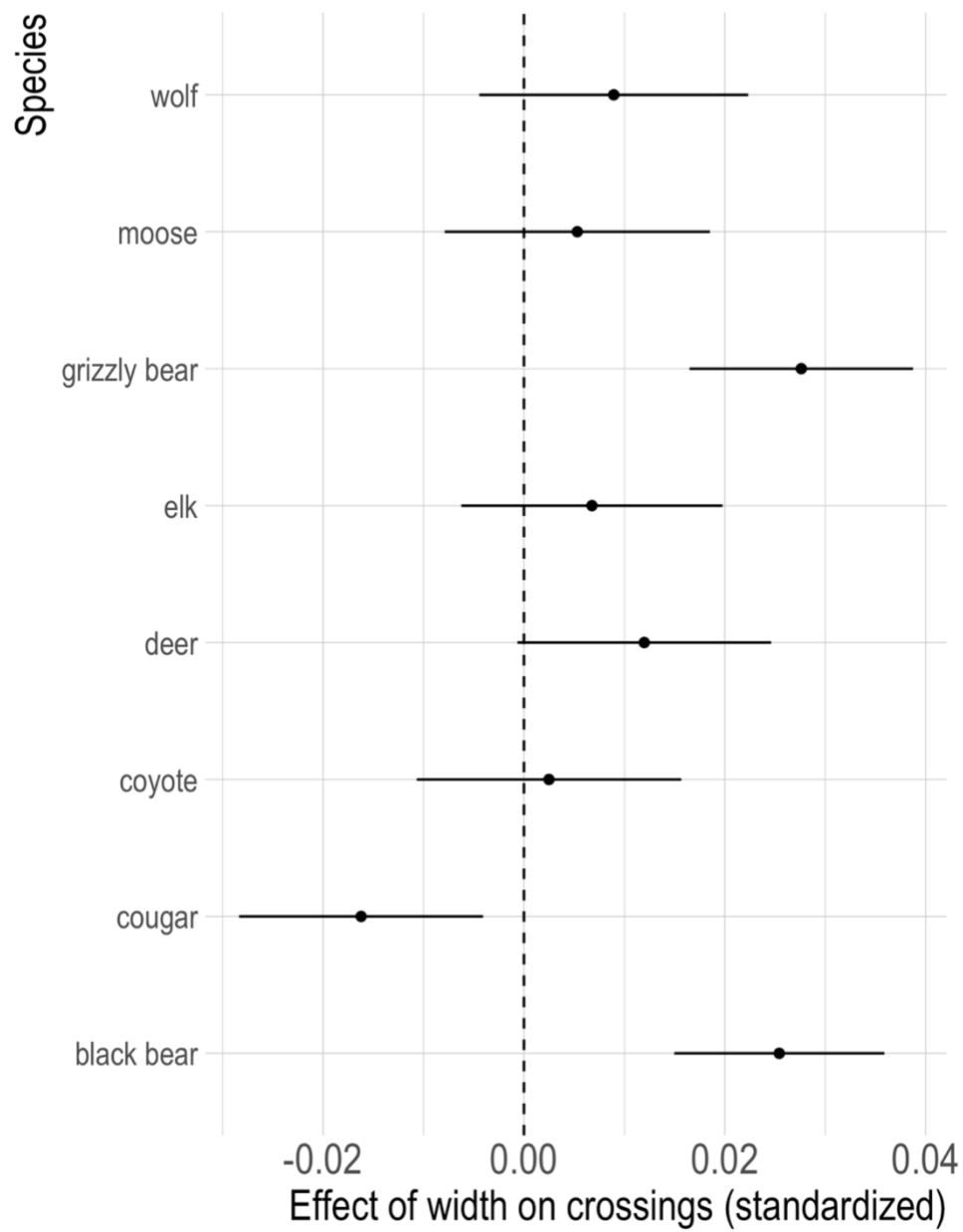

Supplement: Supplemental Information 2 — Error bars are standard errors. Grizzly bear and black bear were significant (p = 0.01497and 0.01706, respectively). Data compiled from transportation agencies and government reports. Species included in analysis: (black bears (Ursus americanus), grizzly bears (Ursus arctos),wolves (Canis lupus), coyote (Canis latrans), cougars (Puma concolor), deer (Odocoileus sp.), elk (Cervus elaphus) moose (Alces alces) crossing rates and the width of 12 overpasses located in western North America. See Supplemental Information Table S7 for details. [file peerj-10-14371-s002.pdf]
