# Supplementary material for: Wildlife overpass structure size, distribution, effectiveness, and adherence to expert design recommendations
Source: PeerJ. 2022 Dec 12;10:e14371. doi: 10.7717/peerj.14371 (PMC9753749; doi:10.7717/peerj.14371)
Supplement: Supplemental Information 3 [file peerj-10-14371-s003.docx]

| **Mean Reported Width^1^ (n = 24)** | 38 m (6-60) |
| --- | --- |
| **Mean Inner Width ^2,3^ (n=97)** | 34 m (3-76) |
| **Mean Outer Width^2,4^ (n=97)** | 39 m (5-99) |
| **Mean Reported Length^1^ (n=18)** | 51 m (20-67) |
| **Mean Roadway Width (measure of length of OP)^2,5^ (n=107)** | 32 m (6-113) |
| **Mean Overpass Headwall Length^2^ (n=90)** | 65 m (21-138) |
| **Mean Overpass Length including approach ramps^2,7^ (n=27)** | 103 m (39-255) |
| **Mean reported W:L^1^ (n=18)** | 0.75 (0.11-1.52) |
| **Mean estimated W: L^2,8^ (n=90)** | 0.58 (0.06-2.76) |
| **Mean Overpass Age (n =79)** | 16 years (3-47) |
| **Mean number of lanes of traffic crossed (n=110)** | 4 (2-8) |

1. *Dimensions found in relevant literature and grey literature*
2. *Dimensions estimated in Google Earth Pro 7.3.4.8573 (64-bit).*
3. *Inner width: the measure of usable surface of the overpass, defined as the inside extent of headwalls or fences as visible from aerial imagery in Google Earth Pro 7.3.4.8573 (64-bit)..*
4. *Outer width: the measure of the lateral extent of the structure including the outermost extent of headwall or fences as visible from aerial imagery in Google Earth Pro 7.3.4.8573 (64-bit)..*
5. *Road width: a measure of the length of the overpass above, defined as the outermost extent of asphalt as visible from aerial imagery in Google Earth Pro 7.3.4.8573 (64-bit).*
6. *Headwall length: a measure of the extent of overpass headwall across the roadway below, in Google Earth Pro 7.3.4.8573 (64-bit).*
7. *Overpass and ramps length: the entire extent of human altered landscape, including earthen ramps (only recorded if visible)*
8. *W:L: width to length ratios using the inner width and headwall length of an overpass*
