# Supplementary material for: Wildlife overpass structure size, distribution, effectiveness, and adherence to expert design recommendations
Source: PeerJ. 2022 Dec 12;10:e14371. doi: 10.7717/peerj.14371 (PMC9753749; doi:10.7717/peerj.14371)
Supplement: Supplemental Information 4 [file peerj-10-14371-s004.docx]

|  | **Global Wildlife Overpass Parameters** | **Expert Recommendations ^3^** | **Compliance** |
| --- | --- | --- | --- |
| **Mean Reported Width (n=20)** | 37 m (6-60) | >50 m | 40% |
| **Mean Reported Length (n=18)** | 51 m (20-67) | - | - |
| **Mean W:L Ratio (n=18)** | 0.75 (0.11-1.52) | ^-^ | - |
| **Mean Overpass Age (n=29)** | 15 years (3-47) | - | - |
