# Supplementary material for: Wildlife overpass structure size, distribution, effectiveness, and adherence to expert design recommendations
Source: PeerJ. 2022 Dec 12;10:e14371. doi: 10.7717/peerj.14371 (PMC9753749; doi:10.7717/peerj.14371)
Supplement: Supplemental Information 5 [file peerj-10-14371-s005.docx]

| **Mean Width (n=28)^1^** | 33 m (6-65) | >50 m | 29% |
| --- | --- | --- | --- |
| **Mean Length (n=27)^2^** | 62 m (29-109) | - | - |
| **Mean W:L Ratio (n=27)** | 0.53 (0.09-1.10) | - | - |
| **Mean Roadway Width (n=28)** | 33 m (10-62) | - | - |
| **Mean Number of Traffic Lanes Crossed (n=28)** | 4 (2-8) | - | - |

1. *Estimated inner width of overpass using Google Earth Pro 7.3.4.8573 (64-bit).*
2. *Estimated headwall length of overpass structures using Google Earth Pro 7.3.4.8573 (64-bit).*
3. *Expert width recommendation of 50m or greater for overpasses in North American (*Clevenger and Huijser*,* 2011)
