# Supplementary material for: Wildlife overpass structure size, distribution, effectiveness, and adherence to expert design recommendations
Source: PeerJ. 2022 Dec 12;10:e14371. doi: 10.7717/peerj.14371 (PMC9753749; doi:10.7717/peerj.14371)
Supplement: Supplemental Information 6 [file peerj-10-14371-s006.docx]

|  | **Global Wildlife Overpass Parameters** | **Expert Recommendations ^3,4^** | **Compliance** |
| --- | --- | --- | --- |
| **Mean Reported Width (n=2)** | 50m | >40 m | 100% |
| **Mean Reported Length (n=0)** | - | - | - |
| **Mean W:L Ratio (n=0)** | - | >0.8 m | - |
| **Mean Overpass Age (n=14)** | 19 years (5-46) | - | - |
