# Supplementary material for: Wildlife overpass structure size, distribution, effectiveness, and adherence to expert design recommendations
Source: PeerJ. 2022 Dec 12;10:e14371. doi: 10.7717/peerj.14371 (PMC9753749; doi:10.7717/peerj.14371)
Supplement: Supplemental Information 7 [file peerj-10-14371-s007.docx]

|  | **Global Wildlife Overpass Parameters** | **Expert Recommendations ^3,4^** | **Compliance** |
| --- | --- | --- | --- |
| **Mean Width (n=52)^1^** | 38m (11-76) | >40 m | 50% |
| **Mean Length (n=52)^2^** | 72 m (23-138) | - | - |
| **Mean W:L Ratio (n=52)** | 0.60 (0.13-2.76) | >0.8 | 21% |
| **Mean Roadway Width (n=51)** | 37 m (8-113) | - | - |
| **Mean Number of Traffic Lanes Crossed (n=51)** | 4 (2-8) | - | - |

1. *Estimated inner width of overpass using Google Earth Pro 7.3.4.8573 (64-bit).*
2. *Estimated headwall length of overpass structures using Google Earth Pro 7.3.4.8573 (64-bit).*
3. *Expert width recommendation of 40m or greater for overpasses in Europe (Iuell, B. (ed.). 2003).*
4. *Expert W:L recommendations of 0.8 or greater for overpasses in Europe (Iuell, B. (ed.). 2003).*
