# Supplementary material for: Wildlife overpass structure size, distribution, effectiveness, and adherence to expert design recommendations
Source: PeerJ. 2022 Dec 12;10:e14371. doi: 10.7717/peerj.14371 (PMC9753749; doi:10.7717/peerj.14371)
Supplement: Supplemental Information 8 [file peerj-10-14371-s008.docx]

|  | **Global Wildlife Overpass Parameters** |
| --- | --- |
| **Mean Reported Width (n = 24)** | 39 m (6-60) |
| **Mean Inner Width ^1^**  **(n= 24)** | 36 m (6-60) |
| **Mean Outer Width^,4^ (n=24)** | 40 m (7-66) |

1. *Estimated inner width of overpass measured with Google Earth Pro 7.3.4.8573 (64-bit).*
2. *Outer width: the measure of the lateral extent of the structure including the outermost extent of headwall or fences as visible from aerial imagery in Google Earth Pro 7.3.4.8573 (64-bit).*
