# Supplementary material for: Wildlife overpass structure size, distribution, effectiveness, and adherence to expert design recommendations
Source: PeerJ. 2022 Dec 12;10:e14371. doi: 10.7717/peerj.14371 (PMC9753749; doi:10.7717/peerj.14371)
Supplement: Supplemental Information 9 — Species included in analysis: (black bears (Ursus americanus), grizzly bears (Ursus arctos),wolves (Canis lupus), coyote (Canis latrans), cougars (Puma concolor), deer (Odocoileus sp.), elk (Cervus elaphus), moose (Alces alces) and bighorn sheep (Ovis canadensis) crossing rates and the width of 12 overpasses located in western North America. [file peerj-10-14371-s009.docx]

| Name | Country | Province/State | Year of build_clean | Width | Length | Width: Length | Number of Monitoring Days | Period of Monitoring | Total Number of Large Mammal Crossings Per Day | Number of large bodied mammal species reported to use (/9) | Source |
| --- | --- | --- | --- | --- | --- | --- | --- | --- | --- | --- | --- |
| Banff National Park Wolverine Overpass | Canada | Alberta | 1996 | 51.62 | 57.72 | 0.89431739 | 3180 | 2006-2014 | 2.15062893 | 8 | (Parks Canada, 2021) |
| Banff National Park Red Earth Overpass | Canada | Alberta | 1996 | 49.48 | 59.51 | 0.8314569 | 3180 | 2007-2015 | 2.78050314 | 8 | (Parks Canada, 2021) |
| Banff National Park Temple Overpass | Canada | Alberta | 2010 | 58.51 | 73.16 | 0.79975396 | 1486 | 2011-2015 | 0.487214 | 7 | (Parks Canada, 2021) |
| Banff National Park Lake Louise Over Pass | Canada | Alberta | 2009 | 59.5 | 69.6 | 0.85488506 | 1471 | 2010-2015 | 0.46838885 | 7 | (Parks Canada, 2021) |
| Banff National Park Castle Overpass | Canada | Alberta | 2011 | 58.05 | 66.29 | 0.87569769 | 1190 | 2011-2015 | 0.90756303 | 8 | (Parks Canada, 2021) |
| Banff National Park Panorama Overpass | Canada | Alberta | 2011 | 57.8 | 67.84 | 0.85200472 | 1203 | 2011-2015 | 0.49293433 | 7 | (Parks Canada, 2021) |
| Trepanier Creek | Canada | British Columbia | 1990 | 5.76 | 56.63 | 0.10171287 | 164 | November 27th 2017 to May 10th 2018 | 0.95121951 | 3 | (BC MOTI, 2021) |
| Glenogle | Canada | British Columbia | 2011 | 6.69 | 47.94 | 0.13954944 | 164 | November 27th 2017 to May 10th 2018 | 0.04268293 | 1 | (BC MOTI, 2021) |
| Golden Hill | Canada | British Columbia | 2011 | 6.51 | 29.12 | 0.22355769 | 164 | November 27th 2017 to May 10th 2019 | 1.76219512 | 5 | (BC MOTI, 2021) |
| Palliser | Canada | British Columbia | 2011 | 6.97 | 35.86 | 0.19436698 | 164 | November 27th 2017 to May 10th 2020 | 0.07317073 | 3 | (BC MOTI, 2021) |
| Highway 93 North | U.S.A | Montana | 2013 | 55.3 | 64.64 | 0.64510347 | 1826 | 1 January 2011 to 31 December 2015 | 3.40963855 | 6 | (Huijser *et al.,* 2016a) |
| Washington OP | U.S.A | Washington | 2018 | 45.64 | 99.26 | 0.45980254 | 1139 | November 2018 to December 2021 | 1.90254609 | 3 | (WYDOT, 2021) |
