# Supplementary material for: Wildlife overpass structure size, distribution, effectiveness, and adherence to expert design recommendations
Source: PeerJ. 2022 Dec 12;10:e14371. doi: 10.7717/peerj.14371 (PMC9753749; doi:10.7717/peerj.14371)
Supplement: Supplemental Information 10 [file peerj-10-14371-s010.docx]

| **Stadium Name** | **Location** | **Length(m) measured using Google Earth path tool** |
| --- | --- | --- |
| Empower Field at Mile High | Denver, CO | 91.8 |
| FedExField | Landover, Maryland | 91.7 |
| FirstEnergy Stadium | Cleveland, OH | 91.5 |
| Heinz Field | Pittsburgh, PA | 91.6 |
| Highmark Stadium | Orchard Park, New York | 91.6 |
| Levi's Stadium | Santa Clara, CA | 91.5 |
| Lumen Field | Seattle, WA | 91.3 |
| MetLife Stadium | East Rutherford, NJ | 92.4 |
| Paul Brown Stadium | Cincinnati, OH | 91.5 |
| Raymond James Stadium | Tampa, FL | 91.6 |
| Soldier Field | Chicago, IL | 91.7 |
| TIAA Bank Field | Jacksonville, FL | 91.7 |
| 3MG Stadium | Orlando, FL | 91.5 |
| Aggie Memorial Stadium | Las Cruces, NM | 91.4 |
| Alaska Airlines Field at Husky Stadium | Seattle, WA | 91.8 |
| Albertsons Stadium | Boise, ID | 90.9 |
| Allen E. Paulson Stadium | Statesboro, GA | 91.6 |
| Alumni Stadium | Chesnut Hill, MA | 91.5 |
| Amon G. Carter Stadium | Fort Worth, TX | 91.7 |
| Apogee Stadium | Denton, TX | 91.8 |
