# Supplementary material for: Wildlife overpass structure size, distribution, effectiveness, and adherence to expert design recommendations
Source: PeerJ. 2022 Dec 12;10:e14371. doi: 10.7717/peerj.14371 (PMC9753749; doi:10.7717/peerj.14371)
Supplement: Supplemental Information 11 [file peerj-10-14371-s011.docx]

**%Error**

= ((sum of measurements / n)-true value) / true value)*100%

= ((91.61-91.44)/91.44) *100%^1^

=0.2%

1. 91.44 = length in meters of American football field
